# The Fascia Lata.—Its Use in Standing at Rest, Etc.

**Published:** 1876-11

**Authors:** Oscar H. Allis

**Affiliations:** Surgeon to the Presbyterian Hospital, Philadelphia, Pa.


### The Fascia Lata; its use in Standing at Rest; its Value in the Diagnosis of Fracture of the Neck of the Femur.

By Oscar H. Allis, M. D., Surgeon to the Presbyterian Hospital, Philadelphia, Pa.

The problem of standing and at the same time placing the muscles of the lower or hinder extremities at rest, is one beautifully wrought out in the higher order of animals. Those whose bodies are near the ground, and upon which the necessity of lying down and rising again entails no inconvenience, have no need of such a rest. But the larger and more useful animals would suffer great inconvenience and great fatigue if they had no other way of resting than by lying down. Take the horse for instance. Farriers relate of some of them that they have never been known to lie down; that neither the bedding nor the body of the animal has shown at any time evidence of his lying, night or day. And, what is quite as remarkable, they say that horses accustomed to lie down will never do so when they are sick; and that when a sick horse lies down it is very likely never to rise again.

The remarkable feature of this will be somewhat cleared up if we will but note the attitude of the horse when *resting.* The fore part of the body lies as in a swing supported by large strong muscles, and when the horse is resting these relax until their fasciæ and sheaths become supporting tendons. A horse is *not so tall* when he is asleep or resting as when awake and active. This may be verified by taking the measurement at the shoulders when he is quiet and at rest, and a second measurement after *waking him up with a blow from the whip.*

But it is to the hind legs of the horse that I would call attention, as there is a strong analogy between the manner of standing at rest in the lower animals and in man. If you notice the horse you will see that he throws the weight of the hinder part of his body on one leg, while he seems to balance himself by resting the other limb on the tip of the hoof. In a few moments he changes his position, throwing the weight upon the other limb. Thus the entire body is rested almost as perfectly and completely as in lying down. Such an arrangement for resting while in the erect posture is possessed by man, though not to the same degree. I have merely directed attention to the most familiar illustration, that in the case of man it might be the better understood.

When we wish to rest ourselves in the erect posture, we first balance the lower extremity upon the foot. This done, we lock the knee-joint *after the fashion of a carriage top hinge.* With the limb balanced on the foot and the knee locked, we cast the body a little toone side, when we experience a sudden arrest of the body. One thing more, and the act is complete. The unoccupied limb is cast a little in front and away from its fellow, as if to poise or balance the body, and we are at rest *absolutely* so far as the must-cles of locomotion are concerned. We remain in this position until a sense of weariness comes over us, when we change the limbs and reverse the attitude. Somesimes we vary this by leaning the body against some firm object, but in all these positions the muscles of locomotion are unemployed. In this *resting* posture a portion of the fascia lata takes the place of the mnscles in sustaining the body, giving the latter the rest we instinctively avail ourselves of.

This fascia lata forms a sheath for all the muscles of the thigh, binding them up in groups, and bringing them into immediate harmony with one another and the femur. Its thinnest portion is at the inner aspect of the thigh where it forms the deep fascia of the adductors. It is much stronger in the she aths formed for the special muscles of locomotion,—*i. e.*, the flexors and extensors. To enablethese muscles to act at greatest advantage, this fascia can be made tense by two strong muscles, the tensor vanginæ femoris and gluteus maximus, so that whenever we stand erect, walk, or run, these two musoles are chiefly concerned in regulating the tension of the femoral aponeurosis. One can easily verify the accuracy of this if while walking or standing he will place his hand upon the out side of the knee, just above the articulation. Here he will find a strong firm tendon, attached to the outer tuberosity of the tibia, that will harden with each step, and become especially prominent.

This tendon-like structure, which is so prominent in the erect but which almost disappears in the sitting posture, is a portion of the fascia lata. If one will carefully trace upward this tendon, he will find that it extends to the crest of the illum, and in its course passes over the great trochanter. This portion of the fascia lata is the strongest, firmest, and thickest of any part of it, and there is a special reason for its great strength. When a person is obliged to stand for any length of time, he finds himself resting on one limb. This resting is in no respect a muscular act, but is accomplished by *pressing the great trochanter against this thickest part of the fascia lata.* If the reader is in the least skeptical upon this point, let him stand up and, resting himself upon one limb, feel the tendon on the outer side of the knee. Let him change to the other limb and see how prominent the corresponding structure becomes. Let him do this quickly, throwing his weight alternately upon one and the other limb, and *notice the suddenness* with which he is arrested. *If this were muscular, the rest would not be so complete. If muscular, the stoppage would not be so sudden. If muscular, I could not verify the experiment upon the cadaver*, as I have often done. All that is necessary is to secure the knee in splints, and the resting attitude can be perfectly counterfeited in the cadaver.

It will thus be seen that when standing *erect*, walking, or running, the act is purely *muscular*, but that man, like the lower animals, has means of resting himself while in the upright position.

This curious and beneficent provision can be turned to good account in fracture of the neck of the thigh. Let the patient stand before you resting his hands upon a table or chair. Notice that his *limbs are parallel, and that both feet rest symmetrically upon the floor.* Now, if there is fracture of the neck, the fascia lata will be tense upon the side of the sound limb, but the *tensor muscles* have no firm *point of resistance* in the fractured one, and cannot make this femoral fascia tense, as in the other limb. Owing to this the examiner will find that the fascia will offer no resistance to an examination of the head of the great trochanter, as it does in the sound state, but is lax, and can be easily indented. He will also notice that the tendon on the outer side of the knee will possess no corresponding prominence with that of the sound limb.

It is necessary that the patient stand while this observation is being tested, for in the erect posture the fascia lata lends its support to the other muscles. In the reclining posture all the muscles are at rest, and hence this feature disappears.

This matter has been under observation for two years, and I am satisfied from repeated verifications that it posses diagnostic value.

In dislocations the limbs can *never* assume parallelism. The injured limb must *always* stand off from its fellow. Hence I have been particular to state that the observer should note that the limbs are parallel, and that both feet rest symmpetrically upon the floor.

One other point of some value. Let the patient lie on his belly. Tell him to press the pelvis into the bed,—*i. e.*, to hug the bed.

In doing this the great gluteus of the sound limb will make a great dimple, but, owing to the shortening of the limb and the want of resistance on the part of the femur, this dimple will be absent on the fractured side.—*Phila. Med. Times.*

